# Associations of pre-pregnancy BMI and gestational weight gain with large-for-gestational-age in women with gestational diabetes mellitus in Southwestern China

**DOI:** 10.1186/s12884-026-09694-x

**Published:** 2026-07-25

**Authors:** Shuhua Qian, Jianqin Yang, Yan Tian, Qian Zhao, Rujuan Wang, Jianjie Yang, Lei Shu, Xinli Zhu, Jiahui He

**Affiliations:** 1 Department of Obstetrics, Obstetrics & Gynecology Hospital of Fudan University, Shanghai Key Lab of Reproduction and Development, Shanghai Key Lab of Female Reproductive Endocrine Related Diseases, Shanghai, 200433 China; 2 Department of Nursing, Yongping County People’s Hospital, Dali, Yunnan China; 3 Department of Emergency, Yongping County People’s Hospital, Dali, Yunnan China; 4 Department of Obstetrics and Gynecology, Yongping County People’s Hospital, Dali, Yunnan China; 5 Department of Neonatology, Obstetrics & Gynecology Hospital of Fudan University, Shanghai Key Lab of Reproduction and Development, Shanghai Key Lab of Female Reproductive Endocrine Related Diseases, Shanghai, 200433 China

**Keywords:** Gestational diabetes mellitus, Large-for-gestational-age, Pre-pregnancy BMI, Gestational weight gain, Sub-high-altitude, Multiethnic population

## Abstract

**Background:**

Large-for-gestational-age (LGA) infants are a common complication of gestational diabetes mellitus (GDM), increasing the risk of obstetric trauma and long-term metabolic disorders. Pre-pregnancy body mass index (ppBMI) and gestational weight gain (GWG) are modifiable maternal factors, but their joint impact on LGA in sub-high-altitude, multiethnic populations remain unclear.

**Objective:**

To examine the independent and combined associations of ppBMI and GWG with LGA risk among women with GDM in a sub-high-altitude, multiethnic region of southwestern China.

**Methods:**

In this retrospective cohort study, clinical data from 407 women with GDM, diagnosed according to the Chinese national guideline (WS 331–2011) at 24–28 weeks of gestation, were analyzed. ppBMI and GWG were categorized according to national criteria. LGA was defined using Chinese birth weight references, with sensitivity analyses applying INTERGROWTH-21st standards. Multivariable logistic regression models evaluated independent and combined associations, adjusting for maternal age, infant sex, and gestational age at delivery. Sensitivity analyses additionally accounted for maternal insulin use, parity, and gravidity.

**Results:**

Women with overweight/obese ppBMI combined with excessive GWG had the highest risk of LGA (adjusted OR 4.03, 95% CI 1.61–10.11). Excessive GWG among normal/underweight women showed a non-significant trend toward increased LGA risk (adjusted OR 2.60, 95% CI 0.95–7.14). Male infant sex was consistently associated with higher LGA odds. Findings were robust across sensitivity analyses and alternative birth weight standards.

**Conclusions:**

Elevated ppBMI combined with excessive GWG is associated with higher LGA risk in women with GDM in sub-high-altitude, multiethnic regions. These findings are exploratory and hypothesis-generating, and may inform hypotheses for future research on BMI-stratified gestational weight patterns and potential intervention strategies. Prospective studies are warranted to confirm these associations and optimize maternal and neonatal outcomes.

**Supplementary Information:**

The online version contains supplementary material available at https://doi.org/10.1186/s12884-026-09694-x.

## Introduction

The global rise in maternal metabolic disorders has become a major public health concern. Gestational diabetes mellitus (GDM) now affects an increasing proportion of pregnancies worldwide and substantially alters fetal growth trajectories. Recent estimates indicate that nearly one in five live births is complicated by hyperglycemia during pregnancy, most attributable to GDM, highlighting its profound implications for maternal and child health [1, 2]. Among the adverse outcomes associated with GDM, the delivery of large-for-gestational-age (LGA) infants represents a significant clinical challenge. Excessive fetal growth increases the risk of obstetric complications, birth trauma, cesarean delivery, and long-term metabolic disorders in offspring [3, 4]. Identifying modifiable determinants of LGA in GDM pregnancies is therefore essential for improving perinatal outcomes.

Fetal growth is a complex process influenced by genetic, nutritional, metabolic, and environmental factors [5]. In GDM, maternal hyperglycemia facilitates excessive transplacental glucose transfer, leading to fetal hyperinsulinemia and accelerated growth, as described by the Pedersen hypothesis [6]. However, glycemic dysregulation alone does not fully explain the heterogeneity in fetal growth among women with GDM. Pre-pregnancy body mass index (ppBMI) and gestational weight gain (GWG) are two influential and potentially modifiable maternal determinants. Elevated ppBMI is consistently associated with insulin resistance, chronic inflammation, and dysregulated lipid metabolism, which may enhance fetal nutrient exposure independent of maternal glucose levels [7]. Excessive GWG has been linked to increased placental mass, altered nutrient transport, and higher risk of fetal overgrowth [8].

Management of ppBMI and GWG relies heavily on clinical guidelines. International recommendations, such as those from the Institute of Medicine (IOM), provide BMI-specific targets for GWG to optimize maternal and neonatal outcomes [9]. However, evidence suggests that the suitability of these targets varies across ethnic groups. Asian populations, in particular, experience higher metabolic risk at lower BMI thresholds than Western populations, raising concerns about the generalizability of existing recommendations [10–12]. Despite numerous studies exploring associations between ppBMI, GWG, and adverse pregnancy outcomes, evidence specifically focused on women with GDM remains limited and inconsistent, especially in non-Western populations.

Existing studies report heterogeneous findings regarding ppBMI, GWG, and LGA risk in GDM pregnancies. Some suggest that elevated ppBMI and excessive GWG independently increase LGA likelihood [13, 14], while others indicate that GWG effects may vary by baseline BMI, glycemic control, or timing of weight gain [15]. Moreover, most studies were conducted in urban, low-altitude, relatively homogeneous populations, limiting generalizability. Environmental factors such as altitude, which may potentially influence placental development, maternal metabolism, and fetal growth, have received little attention [16]. Multiethnic populations may also differ in genetics, diet, and culture, influencing gestational weight dynamics and metabolic risk.

In China, evidence from sub-high-altitude, multiethnic regions among women with GDM is scarce. Critical gaps remain regarding how ppBMI and GWG jointly influence LGA risk and whether current GWG recommendations are appropriate. This study aims to explore associations between ppBMI, GWG, and LGA risk among women with GDM in a sub-high-altitude, multiethnic region of southwestern China. Using clinically collected data from Yongping County People’s Hospital in Yunnan Province, we describe ppBMI and GWG distributions, evaluate independent and combined associations with LGA risk, and examine whether deviations from recommended GWG ranges differ across BMI strata. These analyses are observational and hypothesis-generating, intended to inform future research rather than provide clinical recommendations.

## Materials and methods

### Study design and participants

This retrospective observational study was conducted at Yongping County People’s Hospital, a secondary general hospital located in a sub-high-altitude region of Yunnan Province, southwestern China. Yongping County is situated in a mountainous area at an average altitude of approximately 1,800 m above sea level. Regions at 1,500–2,500 m are commonly classified as sub-high-altitude areas. The study setting is characterized by moderate altitude and a multiethnic population, providing a distinct environmental and demographic context. Clinical data were collected from women who delivered between January 1, 2022 and December 1, 2025. The study examined associations between ppBMI, GWG, and the risk of LGA infants among women with GDM.

Eligible participants were identified from the hospital obstetric database. Inclusion criteria were: (1) diagnosis of GDM during pregnancy; (2) delivery at the study hospital during the specified period; (3) singleton pregnancy; (4) term delivery; and (5) complete clinical and anthropometric data. Exclusion criteria included pregestational diabetes, severe maternal comorbidities, multiple gestation, fetal congenital anomalies or chromosomal abnormalities, severe placental or umbilical cord abnormalities, fetal growth restriction, stillbirth, or intrauterine fetal death.

**Fig. 1:**
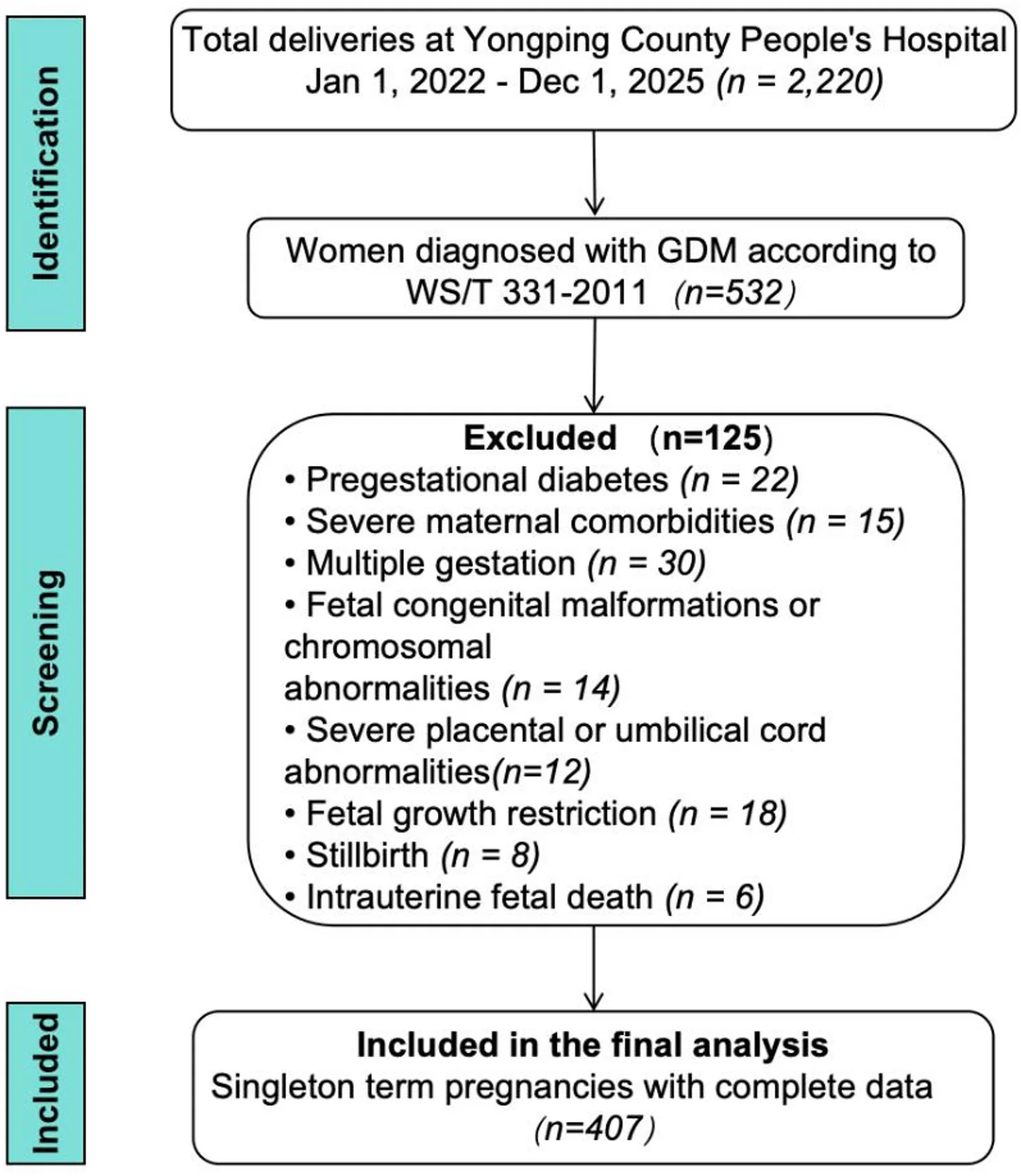
Flowchart of participant selection

After applying these criteria, 407 women with GDM were included in the final analysis. The participant selection process is shown in Fig. 1. As all eligible cases within the study period were included, no formal a priori sample size calculation was performed. Some subgroups, such as underweight women with inadequate GWG, were small, which may have limited statistical power and contributed to marginal findings in certain analyses.

### GDM Diagnosis and management

GDM was diagnosed according to the Chinese national guideline (WS/T 331–2011) [17]. All participants underwent a 75-g oral glucose tolerance test (OGTT) between 24 and 28 weeks of gestation. Plasma glucose levels were measured at fasting, 1 h, and 2 h. GDM was diagnosed if one or more of the following thresholds were met: fasting glucose ≥ 5.1 mmol/L, 1-hour glucose ≥ 10.0 mmol/L, or 2-hour glucose ≥ 8.5 mmol/L. Women diagnosed with GDM received standard clinical management, including medical nutrition therapy, lifestyle counseling, and insulin therapy when indicated. Glycemic control was monitored using fasting and postprandial blood glucose measurements during routine prenatal visits.

### Definitions of exposure, outcome, and covariates

Maternal blood samples for GDM diagnosis were collected in the morning after an overnight fast of at least 8 h. Plasma glucose was measured using the standard hospital laboratory procedures. Blood samples were processed immediately or stored at 4 °C for short-term analysis and at − 20 °C for longer-term storage, following routine clinical protocols. Pre-pregnancy weight was self-reported by participants at the first prenatal visit and recorded in the medical record. Height was measured during the first antenatal examination using standardized equipment. PpBMI was calculated as pre-pregnancy weight divided by height squared (kg/m²). BMI categories were defined according to the Chinese adult BMI classification criteria as follows: underweight (< 18.5 kg/m²), normal weight (18.5–23.9 kg/m²), overweight (24.0–27.9 kg/m²), and obesity (≥ 28.0 kg/m²) [18]. For regression analyses, ppBMI categories were further collapsed into two groups (underweight/normal weight and overweight/obese) to ensure adequate statistical power and stable model estimates. GWG was calculated as the difference between maternal weight at delivery and pre-pregnancy weight. Maternal weight during pregnancy was measured at routine prenatal visits using standardized clinical scales by trained healthcare staff. The final pregnancy weight was obtained from the measurement recorded at hospital admission for delivery. GWG was classified as inadequate, adequate, or excessive according to the national Chinese guideline for weight gain during pregnancy (WS/T 801–2022), issued by the National Health Commission of China [19]. This standard provides BMI-specific recommended ranges for gestational weight gain and is widely used in clinical practice and perinatal research in China.

The primary outcome was LGA. LGA was defined as birth weight above the 90th percentile for gestational age and sex, using the national Chinese birth weight reference standards (WS/T 800–2022), which were developed from a large multicenter survey and are recommended for perinatal growth assessment in Chinese populations [20]. To assess the robustness of the findings, LGA classification was also evaluated using the INTERGROWTH-21st international newborn size standards, which provide global reference curves for birth weight by gestational age and sex based on a multi-country study [21]. LGA was chosen as the outcome measure instead of macrosomia because it accounts for gestational age–specific fetal growth and more accurately reflects excessive intrauterine growth across different gestational weeks. Covariates included infant sex, gestational age at delivery, and maternal age. These variables were selected based on established clinical relevance to fetal growth and birth weight, supported by prior literature and biological plausibility. Additional potential confounders, such as maternal insulin use, gravidity, and parity, were not included in the primary model due to the limited number of LGA events and the risk of overfitting, in accordance with the events-per-variable (EPV) principle described in the Statistical Analysis section. These variables were instead examined individually in sensitivity analyses. Despite these approaches, residual confounding cannot be completely excluded.

### Statistical analysis

Continuous variables were summarized as mean ± standard deviation, and categorical variables were presented as numbers and percentages. Differences between LGA and non-LGA groups were assessed using the χ² test or Fisher’s exact test for categorical variables and the Mann–Whitney U test for continuous variables with non-normal distribution. Univariable binary logistic regression analyses were performed to estimate unadjusted odds ratios and 95% confidence intervals for factors associated with LGA. Multivariable logistic regression models were then constructed to evaluate the associations of ppBMI and GWG with LGA risk. Model 1 included ppBMI as the main exposure, Model 2 included GWG, and Model 3 evaluated LGA risk across different combinations of ppBMI and GWG categories without formally testing statistical interaction. All multivariable models were adjusted for infant sex, gestational age at delivery, and maternal age. To maintain model stability and reduce overfitting, the number of covariates was restricted according to the EPV principle, with a minimum of approximately 10 events per variable [22]. With four variables in the primary models, the EPV was approximately 11.8, which is generally considered adequate for logistic regression. To address potential bias due to quasi-complete separation in certain subgroups, a sensitivity analysis using Firth penalized logistic regression was additionally performed. Post hoc power analysis was not performed. Adjusted odds ratios with 95% confidence intervals were reported. A two-sided P value < 0.05 was considered statistically significant. All statistical analyses were performed using SPSS software (version 31.0; IBM Corp., Armonk, NY, USA), with the Firth penalized logistic regression conducted using R software (version 4.5.3; R Foundation for Statistical Computing, Vienna, Austria). Forest plots of adjusted odds ratios were generated using GraphPad Prism version 10.6.

## Results

### Participant characteristics

A total of 407 women with GDM who delivered term singleton infants were included in the analysis. Among them, 47 (11.5%) delivered LGA infants and 360 (88.5%) delivered non-LGA infants. Baseline characteristics stratified by LGA status are presented in Table 1. Women who delivered LGA infants had a significantly higher proportion of excessive GWG (65.60% vs. 35.56%, *P* < 0.001) and a greater proportion of male infants (68.09% vs. 52.78%, *P* = 0.048), indicating that excessive GWG and male fetal sex were more common among LGA births. No significant differences were observed between groups in maternal age, ethnicity, ppBMI category, gestational age at delivery, education level, or occupation (all *P* > 0.05).

**Table 1:** Baseline characteristics of women with GDM according to LGA status

Variable	Non-LGA (*n* = 360 )	LGA (*n* = 47 )	*P* value
Maternal age (years)			0.266
< 25	67 (18.61)	5 (10.64)	
25–35	232(64.44)	36(76.60)	
> 35	61 (16.94)	6(12.77)	
Ethnicity			0.669
Han	227(63.06)	31(65.96)	
Yi	77(21.39)	11(23.40)	
Other minority groups	56 (15.55)	5(10.64)	
Pre-pregnancy BMI category			0.280
Underweight	16(4.44)	0 (0.00)	
Normal weight	161 (44.72)	18 (38.30)	
Overweight	114 (31.67)	16 (34.04)	
Obese	69 (19.17)	13(27.66)	
Gestational weight gain (GWG)			< 0.001
Inadequate	55 (15.28)	3(6.39)	
Adequate	177(49.17)	13 (27.66)	
Excessive	128 (35.56)	31(65.60)	
Gestational age at delivery (weeks)	39.02 ± 0.95	39.13 ± 0.77	0.628
Infant sex			0.048
Male	190 (52.78)	32 (68.09)	
Female	170 (47.22)	15(31.91)	
Education level			0.423
Junior high school or below	101(28.06)	9(19.15)	
Technical secondary school or senior high school	118(32.78)	18(38.30)	
College or university	141(39.17)	20(42.55)	
Occupation			0.599
Farmer	287(79.72)	39(82.98)	
Non-agricultural occupation	73(20.28)	8(17.02)	
Mode of delivery			< 0.001
Cesarean section	201(55.83)	39(82.98)	
Vaginal delivery	159(44.17)	8(17.02)	

Data are presented as n (%) for categorical variables and mean ± SD for continuous variables. P values were calculated using χ² test or Fisher’s exact test for categorical variables and Mann-Whitney U test for gestational age (non-normal distribution in Non-LGA group). Other minority group includes Hui (*n* = 22), Bai (*n* = 27), Lisu (*n* = 6), Naxi (*n* = 1), Jingpo (*n* = 1), Gelao (*n* = 1), Achang(*n* = 2), and Miao (*n* = 1)

## Univariable associations with LGA

Univariable logistic regression analyses examining factors associated with LGA are presented in Table 2. Excessive GWG was significantly associated with increased LGA risk compared with adequate GWG (crude OR 3.30, 95% CI 1.66–6.55, *P* = 0.001), suggesting that women with excessive GWG had more than threefold higher odds of delivering an LGA infant. Male infant sex showed a borderline significant association (crude OR 1.91, 95% CI 1.00–3.65, *P* = 0.050), corresponding to approximately doubled odds of LGA compared with female infants. Overweight/obese ppBMI (vs. underweight/normal) demonstrated a trend toward higher risk, although not statistically significant (crude OR 1.56, 95% CI 0.84–2.91, *P* = 0.163), suggesting a possible moderate increase in risk, although the association did not reach statistical significance. No significant univariable associations were found for maternal age categories, ethnicity, inadequate GWG, occupation, education level, or gestational age at delivery (all *P* > 0.05).

**Table 2:** Unadjusted odds ratios for risk factors associated with LGA infants

Variable	Category	OR	95% CI	*P* value
Infant sex	Male	1.91	1.00–3.65	0.050
	Female	Ref	–	-
Maternal age (years)	< 25 vs. 25–35	0.48	0.18–1.27	0.141
	> 35 vs. 25–35	0.63	0.26–1.57	0.326
	25–35	Ref	-	-
Ethnicity	Minority	0.88	0.46–1.67	0.698
	Han	Ref	-	-
PpBMI	Overweight/Obese	1.56	0.84–2.91	0.163
	Underweight/Normal	Ref	-	-
GWG	Inadequate	0.74	0.20–2.70	0.652
	Excessive	3.30	1.66–6.55	0.001
	Adequate	Ref	-	-
Occupation	Non-agricultural occupation	0.81	0.36–1.80	0.600
	Farmer	Ref	-	-
Education level	Junior high school or below	0.63	0.28–1.44	0.271
	Technical secondary school or senior high school	1.08	0.54–2.13	0.834
	College or university	Ref	-	-
Gestational age at delivery (weeks)		1.14	0.82–1.58	0.454

Univariable binary logistic regression analysis

*BMI *body mass index, *CI *confidence interval, *GWG *gestational weight gain, *OR *odds ratio, *Ref *reference category

### Multivariable associations with LGA

Goodness-of-fit for all multivariable logistic regression models was assessed using the Hosmer–Lemeshow test, with results indicating acceptable fit (Model 1: χ² = 4.456, df = 8, *P* = 0.814; Model 2: χ² = 12.854, df = 8, *P* = 0.117; Model 3: χ² = 8.745, df = 8, *P* = 0.364). Model fit was modest, with Nagelkerke R² values of 0.033 for Model 1, 0.094 for Model 2, and 0.114 for Model 3. No formal correction for multiple testing was performed, as the analyses were hypothesis-driven and limited to a small number of clinically relevant exposures.

**Fig. 2:**
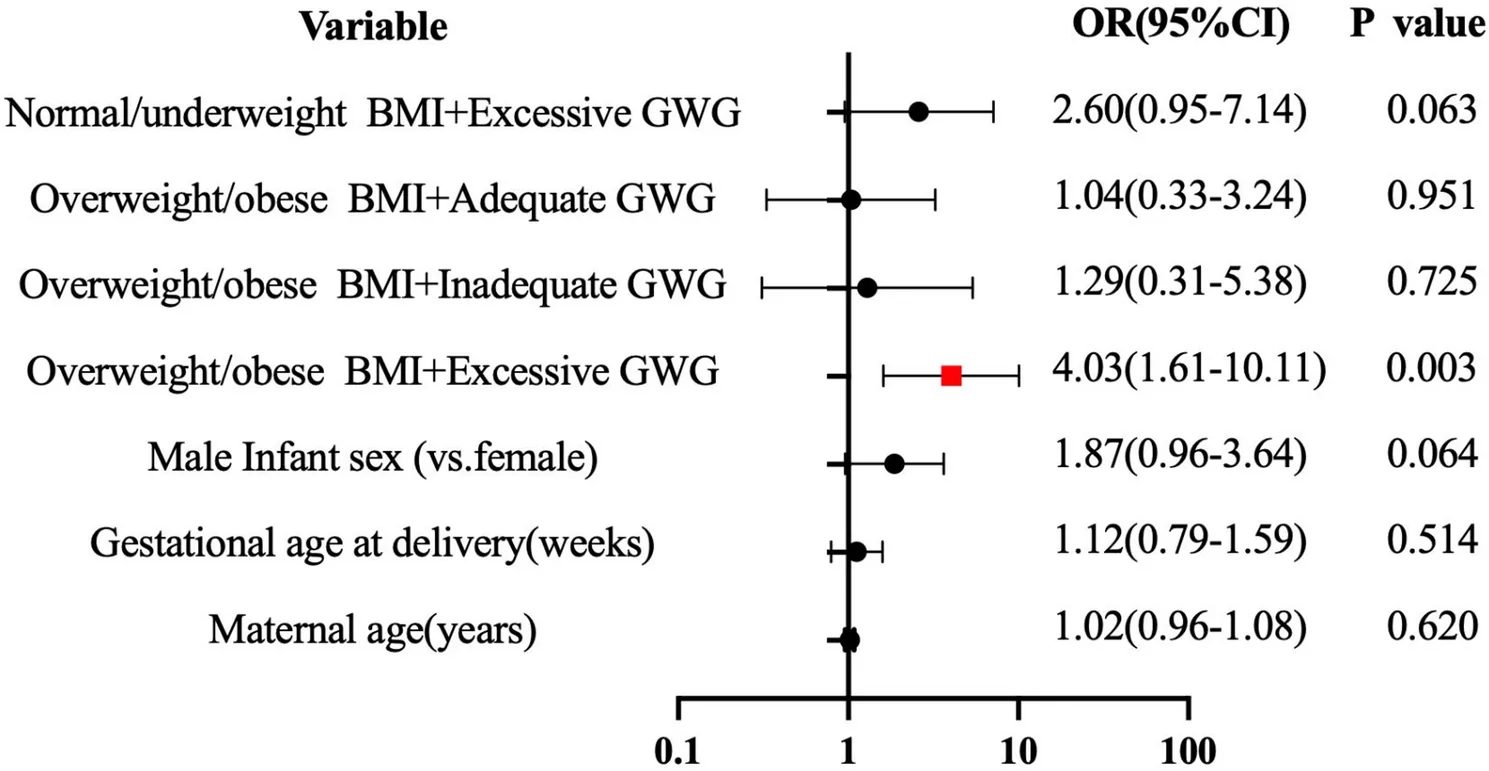
Forest plot of adjusted odds ratios (ORs) and 95% confidence intervals (CIs) for LGA infants based on multivariable logistic regression (Model 3). Notes: a The reference group was normal/underweight BMI with adequate GWG. b Odds ratios are plotted on a logarithmic scale to allow symmetric visualization of effects above and below 1. c Error bars represent 95% confidence intervals. d The subgroup with no events (Normal/underweight BMI + Inadequate GWG) was not plotted due to quasi-complete separation; corresponding OR could not be estimated. e Infant sex (male vs. female), maternal age (years), and gestational age at delivery (weeks) were included as covariates in all models

Multivariable logistic regression models, adjusted for infant sex, maternal age and gestational age at delivery, were used to assess the independent and combined effects of ppBMI and GWG on LGA risk (Table 3). In Model 1, overweight/obese ppBMI was associated with a non-significant increase in LGA risk (adjusted OR 1.60, 95% CI 0.85–3.01, *P* = 0.144), indicating approximately 60% higher odds of LGA compared with underweight/normal BMI, although this did not reach statistical significance. In Model 2, excessive GWG remained strongly and independently associated with higher LGA risk (adjusted OR 3.33, 95% CI 1.67–6.64, *P* = 0.001), indicating more than threefold increased odds. The magnitude of this association indicates that excessive GWG may represent a potentially important factor associated with LGA risk among women with GDM. Inadequate GWG showed no significant association (adjusted OR 0.75, 95% CI 0.21–2.76, *P* = 0.668). Model 3 evaluated LGA risk across different combinations of ppBMI and GWG categories. Compared with the reference category (underweight/normal ppBMI with adequate GWG), the combination of overweight/obese ppBMI and excessive GWG was associated with the highest LGA risk (adjusted OR 4.03, 95% CI 1.61–10.11, *P* = 0.003), corresponding to more than fourfold higher odds. This finding indicates a notable combined association of pre-pregnancy overweight/obesity and excessive weight gain during pregnancy. Excessive GWG in the context of underweight/normal ppBMI also showed a trend toward increased risk (adjusted OR 2.60, 95% CI 0.95–7.14, *P* = 0.063), representing more than doubled odds, although statistical significance was not achieved. Other combinations did not reach statistical significance. This finding suggests that the association between excessive GWG and LGA may be stronger among women with pre-pregnancy overweight/obesity. Male infant sex demonstrated a consistent borderline association across models (adjusted OR 1.87–1.95, *P* = 0.045–0.064), indicating approximately 80% to 90% higher odds of LGA compared with female infants. Gestational age at delivery (adjusted OR ≈ 1.12–1.13 per week, *P* > 0.483) and maternal age (adjusted OR ≈ 1.01–1.02 per year, *P* = 0.549–0.861) had no significant effect. Forest plots illustrating the adjusted odds ratios from Model 3 are shown in Fig. 2. Across combined exposure categories, the highest LGA risk was observed among women with overweight/obese BMI and excessive GWG, with more than fourfold increased odds compared with the reference group (underweight/normal BMI with adequate GWG). Excessive GWG among women with underweight/normal BMI also showed a trend toward increased risk, while other combinations were not significantly associated with LGA.

**Table 3:** Multivariable adjusted odds ratios for LGA infants

Variable	Category	Model 1 Adjusted OR (95% CI)	*P* value	Model 2 Adjusted OR (95% CI)	*P* value	Model 3 Adjusted OR (95% CI)	*P* value
PpBMI	Overweight/obese	1.60 (0.85–3.01)	0.144				
	Underweight/normal	Ref	–				
GWG	Inadequate			0.75 (0.21–2.76)	0.668		
	Excessive			3.33 (1.67–6.64)	0.001		
	Adequate			Ref	–		
PpBMI × GWG	Normal/underweight BMI + Adequate GWG					Ref	–
	Normal/underweight BMI + Inadequate GWG					*	0.998
	Normal/underweight BMI + Excessive GWG					2.60 (0.95–7.14)	0.063
	Overweight/obese BMI + Adequate GWG					1.04 (0.33–3.24)	0.951
	Overweight/obese BMI + Inadequate GWG					1.29(0.31–5.38)	0.725
	Overweight/obese BMI + Excessive GWG					4.03 (1.61–10.11)	0.003
Infant sex	Male	1.95 (1.02–3.74)	0.045	1.89 (0.98–3.66)	0.059	1.87 (0.96–3.64)	0.064
	Female	Ref	–	Ref	–	Ref	–
Gestational age at delivery (weeks)	Continuous (per 1-week increase)	1.13 (0.80–1.59)	0.483	1.12 (0.79–1.59)	0.505	1.12 (0.79–1.59)	0.514
Maternal age (years)	Continuous (per 1-year increase)	1.01(0.95–1.07)	0.861	1.02(0.96–1.08)	0.549	1.02(0.96–1.08)	0.620

All models adjusted for infant sex, maternal age and gestational age at delivery (continuous, per 1-week increase)

Model 1 included ppBMI as the main exposure; Model 2 included GWG as the main exposure; Model 3 included combined ppBMI and GWG patterns as the main exposure

*aOR *adjusted odds ratio, *CI *confidence interval, *Ref  *reference category

* Estimate unreliable due to quasi-complete separation; no LGA events occurred in this subgroup, leading to model non-convergence and unstable odds ratio estimates. Interpret this value with caution

### Sensitivity analysis

Reclassification of LGA using the INTERGROWTH-21st international standards identified 35 cases (8.6%) compared with 47 cases (11.5%) in the primary analysis. Multivariable logistic regression models were re-run to assess the robustness of the main findings (Table 4). Excessive gestational weight gain remained significantly associated with LGA risk (adjusted OR 2.87, 95% CI 1.31–6.28, *P* = 0.008 in Model 2), indicating a consistent direction and magnitude of association when the international growth standard was applied. In Model 3, the combined effect of pre-pregnancy overweight/obesity and excessive GWG was attenuated, with the highest risk observed for overweight/obese BMI with excessive GWG (adjusted OR 2.55, 95% CI 0.98–6.68, *P* = 0.056), corresponding to more than doubled odds, although statistical significance was not achieved. The reduction in effect size compared with the primary analysis may be related to the smaller number of LGA cases under the alternative classification, which could have limited statistical precision. Despite these differences, the direction and overall magnitude of the associations remained similar to the primary analysis, supporting the consistency of the findings across analytical approaches. Additional sensitivity analyses were conducted to further evaluate the robustness of Model 3 by individually adjusting for maternal insulin use, gravidity, and parity. As shown in Supplementary Tables S1, the associations between pre-pregnancy BMI, gestational weight gain, and LGA remained largely unchanged. Furthermore, sensitivity analysis using Firth penalized logistic regression yielded stable estimates and results consistent with the primary analysis (Supplementary Table S3). Overall, these findings indicate that the observed associations were stable across multiple analytical strategies.

**Table 4:** Sensitivity analysis of key multivariable associations with LGA using INTERGROWTH-21st standards

Model	Variable / Category	Primary Analysis Adjusted OR (95% CI)	*P* value	Sensitivity Analysis Adjusted OR (95% CI)	*P* value
Model 2	Excessive GWG vs. Adequate GWG	3.33 (1.67–6.64)	0.001	2.87 (1.31–6.28)	0.008
Model 3	Normal/underweight BMI + Excessive GWG vs. Reference*	2.60 (0.95–7.14)	0.063	1.74 (0.60–5.07)	0.312
Model 3	Overweight/obese BMI + Excessive GWG vs. Reference*	4.03 (1.61–10.11)	0.003	2.55 (0.98–6.68)	0.056

*Reference category: normal/underweight ppBMI with adequate GWG. All models adjusted for infant sex and gestational age at delivery. Sensitivity analysis used LGA defined according to the INTERGROWTH-21st international newborn standards. The slightly lower effect estimates in Model 3 reflect fewer events in some categories but do not alter the overall interpretation

## Discussion

This study examined the associations between ppBMI, GWG, and the risk of LGA infants among women with GDM in a sub-high-altitude, multiethnic region of southwestern China. Excessive GWG was associated with an increased risk of LGA. This association appeared stronger among women who were overweight or obese before pregnancy, with an adjusted odds ratio of 4.05. These findings suggest that modifiable maternal factors may influence fetal growth patterns in GDM, particularly in populations exposed to environmental stressors such as sub-high-altitude conditions. They also highlight the need for further research into mechanisms underlying intergenerational metabolic risk.

The increased LGA risk associated with excessive GWG, especially among women with higher ppBMI, may reflect additive effects rather than formally tested statistical interactions [23]. In GDM, maternal hyperglycemia can induce fetal hyperinsulinemia, promoting adipose tissue accumulation and organ growth via insulin and insulin-like growth factor pathways, as proposed by the Pedersen hypothesis [6]. Excessive GWG may further exacerbate fetal overnutrition by influencing placental nutrient transport, although this mechanism remains speculative in the absence of direct placental or hypoxia measurements [24]. Pre-existing insulin resistance in women with elevated ppBMI may also reduce maternal nutrient utilization, increasing substrate transfer to the fetus [25, 26]. The stronger association observed among women with both overweight/obesity and excessive GWG suggests a potential threshold-dependent additive effect, though formal interaction testing was not conducted. The borderline association with male infant sex may reflect sex-specific placental responses, whereas gestational age showed no significant association, consistent with standardized LGA definitions for term births [27–29]. These interpretations are exploratory and intended to generate hypotheses for future research.

To address potential instability due to sparse data in certain exposure categories, we conducted sensitivity analyses using Firth penalized logistic regression. The results were consistent with the primary analyses, with the combination of overweight or obese ppBMI and excessive GWG remaining associated with the highest odds of LGA. This supports the relative stability of the observed associations.

Comparison with existing literature indicates general agreement with prior findings while highlighting the unique contributions of this study. Excessive GWG has been consistently linked to increased LGA risk across diverse populations, including meta-analyses involving over one million pregnancies [30]. Additive effects of ppBMI and GWG have also been observed in GDM populations [31]. In Chinese cohorts, elevated ppBMI and excessive GWG have been identified as major contributors to LGA, with BMI-stratified models emphasizing their combined influence [32]. Longitudinal studies further suggest that excessive GWG is associated with adverse offspring growth and long-term metabolic outcomes [23, 33]. Variations across studies may reflect differences in population characteristics and environmental contexts. For example, in some European populations without GDM, ppBMI appears more strongly associated with LGA than GWG, potentially underestimating combined effects in sub-high-altitude regions [34]. Lifestyle factors, including physical activity, may also modify the impact of excessive GWG among women with obesity and early GDM. In the present cohort, the multiethnic composition and potential diversity in diet and genetics may influence metabolic responses [35]. Differences in GWG classification standards may also contribute to inconsistent findings. The absence of a protective effect of inadequate GWG in this study may relate to the use of WS/T 801–2022 criteria, which differ from IOM recommendations and are tailored to Asian populations [36, 37]. Regional differences in BMI thresholds may further influence risk classification [38]. These considerations underscore the importance of environmental and ethnic context when interpreting findings and highlight the relevance of this study to underrepresented populations [39].

These findings may have potential theoretical and research implications. Although mechanistic explanations related to sub-high-altitude hypoxia remain speculative, they may inform future studies on fetal programming. The results support further investigation into stratified approaches to maternal weight management, including targeted monitoring or counseling for women with GDM who are overweight or obese. Future studies in sub-high-altitude, multiethnic settings may also explore altitude-adapted nutritional strategies and individualized care approaches. At the population level, optimizing ppBMI may be relevant for long-term metabolic health, although feasibility and resource considerations require further evaluation.

In summary, the combined presence of overweight or obesity before pregnancy and excessive GWG was associated with increased LGA risk. These findings highlight potential directions for research on individualized weight management and emphasize the importance of contextual factors, including environment and ethnicity. Future studies should include prospective designs and interventional trials, as well as integrative approaches such as multi-omics, to better understand altitude–ethnicity interactions and perinatal outcomes. Given the observational design and potential residual confounding, the findings should be interpreted as exploratory and are not intended to directly guide clinical practice.

### Limitations

Several limitations should be acknowledged. First, the retrospective design and reliance on medical records, including self-reported pre-pregnancy weight, may introduce misclassification bias and preclude causal inference. Second, the relatively small sample size limited statistical power in some subgroups and led to quasi-complete separation in rare exposure categories, although key findings were consistent in sensitivity analyses. Third, important confounders, such as detailed glycemic control, GDM management strategies, lifestyle behaviors, and socioeconomic factors, were not available, and residual confounding cannot be excluded. Fourth, the inclusion of both Han and minority ethnic groups introduces heterogeneity related to diet, physical activity, and cultural practices, which were not directly measured. Fifth, GWG by specific gestational periods was not analyzed, limiting assessment of temporal patterns. Sixth, additional maternal and neonatal outcomes, such as shoulder dystocia and neonatal hypoglycemia, were not systematically captured. Finally, the restriction to term singleton pregnancies may limit generalizability to higher-risk populations.

## Conclusions

In this retrospective cohort of women with GDM in a sub-high-altitude, multiethnic region of southwestern China, excessive gestational weight gain was associated with an increased risk of LGA, particularly among women who were overweight or obese before pregnancy. These associations were consistent across multiple sensitivity analyses, including alternative growth standards and additional covariate adjustments. However, given the observational design, causal relationships cannot be established. The findings should be interpreted as exploratory and hypothesis-generating, providing a basis for future research rather than direct clinical recommendations. Prospective studies and interventional trials are needed to confirm these associations and to evaluate whether targeted strategies, such as individualized nutritional counseling, lifestyle modification, and weight monitoring, can influence LGA risk in GDM pregnancies.

## Supplementary Information

Supplementary Material 1.

## Data Availability

Clinical data were collected from anonymized medical records of women who delivered between January 1, 2022, and December 1, 2025, at Yongping County People’s Hospital. These data were used to examine the associations between ppBMI, GWG, and the risk of LGA infants in women with GDM. Due to patient confidentiality regulations, the raw datasets are not publicly available. De-identified data supporting the findings of this study can be obtained from the corresponding author upon reasonable request, subject to approval by the hospital’s Ethics Committee.
