# Supplementary material for: Associations of pre-pregnancy BMI and gestational weight gain with large-for-gestational-age in women with gestational diabetes mellitus in Southwestern China
Source: BMC Pregnancy Childbirth. 2026 Jul 25;26:1206. doi: 10.1186/s12884-026-09694-x (PMC13688827; doi:10.1186/s12884-026-09694-x)
Supplement: Supplementary file 1 [file 12884_2026_9694_MOESM1_ESM.docx]

**Supplementary Tables S1. Sensitivity Analysis: Multivariable Adjusted Odds Ratios for LGA Infants**

| **Variable** | **Category** | **Model 3 Original OR (95% CI)** | **P value** | **Model 3 + Maternal insulin OR (95% CI)** | **P value** | **Model 3 + Parity OR (95% CI)** | **P value** | **Model 3 + Gravidity OR (95% CI)** | **P value** |  |  |  |
| --- | --- | --- | --- | --- | --- | --- | --- | --- | --- | --- | --- | --- |
| Pre-pregnancy BMI × GWG | Normal/underweight BMI + Adequate GWG | Ref | – | Ref | – | Ref | – | Ref | – |  |  |  |
|  | Normal/underweight BMI + Inadequate GWG | * | 0.998 | * | 0.998 | * | 0.998 | * | 0.998 |  |  |  |
|  | Normal/underweight BMI + Excessive GWG | 2.60 (0.95–7.14) | 0.063 | 2.60 (0.95–7.14) | 0.063 | 2.63 (0.96–7.24) | 0.061 | 2.59 (0.95–7.12) | 0.064 |  |  |  |
|  | Overweight/obese BMI + Adequate GWG | 1.04 (0.33–3.24) | 0.951 | 1.04 (0.33–3.28) | 0.942 | 1.04 (0.33–3.24) | 0.953 | 1.03 (0.33–3.22) | 0.959 |  |  |  |
|  | Overweight/obese BMI + Inadequate GWG | 1.29(0.31–5.38) | 0.725 | 1.30 (0.31–5.42) | 0.720 | 1.28 (0.31–5.34) | 0.736 | 1.27 (0.30–5.30) | 0.743 |  |  |  |
|  | Overweight/obese BMI + Excessive GWG | 4.03 (1.61–10.11) | 0.003 | 4.02 (1.60–10.10) | 0.003 | 4.03 (1.61–10.13) | 0.003 | 3.98 (1.58–10.00) | 0.003 |  |  |  |
| Infant sex | Male | 1.87 (0.96–3.64) | 0.064 | 1.87 (0.96–3.64) | 0.064 | 1.88 (0.97–3.65) | 0.063 | 1.86 (0.96–3.63) | 0.066 |  |  |  |
| Gestational age at delivery (weeks) | Continuous | 1.12 (0.79–1.59) | 0.514 | 1.12 (0.79–1.59) | 0.519 | 1.12 (0.79–1.59) | 0.509 | 1.13 (0.80–1.60) | 0.505 |  |  |  |
| Maternal age(years) | Continuous | 1.02(0.96-1.08) | 0.620 | 1.02(0.96-1.08) | 0.621 | 1.01(0.94-1.08) | 0.758 | 1.01(0.94-1.08) | 0.841 |  |  |  |
| Maternal insulin use | Yes vs No | – | – | 0.87 (0.11–7.19) | 0.898 | – | – | – | – |  |  |  |
| Parity | Continuous | – | – | – | – | 1.07 (0.63–1.82) | 0.805 | – | – |  |  |  |
| Gravidity | Continuous | – | – | – | – | – | – | 1.08 (0.82–1.42) | 0.595 |  |  |  |

Notes:

a. The original Model 3 adjusted for infant sex, maternal age, and gestational age at delivery (continuous, per 1-week increase). Sensitivity analyses additionally adjusted individually for maternal insulin use, parity, or gravidity.

b. The reference category was normal/underweight BMI with adequate gestational weight gain (GWG).

c. * Estimate unreliable due to quasi-complete separation; no LGA events occurred in this subgroup, leading to model non-convergence and unstable odds ratio estimates. Interpret this value with caution.

d. OR = odds ratio; CI = confidence interval; Ref = reference category.

**Supplementary Table S2. Distribution of LGA events across combined prepregnancy BMI and GWG categories**

| **Prepregnancy BMI category** | **GWG category** | **Total (n)** | **LGA (n)** | **LGA(%)** |
| --- | --- | --- | --- | --- |
| Normal/underweight | Adequate | 102 | 7 | 6.9 |
| Normal/underweight | Inadequate | 23 | 0 | 0 |
| Normal/underweight | Excessive | 70 | 11 | 15.7 |
| Overweight/obese | Adequate | 88 | 6 | 6.8 |
| Overweight/obese | Inadequate | 35 | 3 | 8.6 |
| Overweight/obese | Excessive | 89 | 20 | 22.5 |
| Total |  | 407 | 47 | 11.5 |

Notes:

BMI = body mass index; GWG = gestational weight gain; LGA = large-for-gestational-age. LGA was defined according to the WS/T 800–2022 birthweight reference. The category “normal/underweight BMI + inadequate GWG” contained no LGA events, which resulted in quasi-complete separation and prevented reliable estimation of the odds ratio in standard logistic regression models.

**Supplementary Table S3. Sensitivity Analysis Using Firth Penalized Logistic Regression (Model 3)**

| **Variable** | **Category** | **aOR (95% CI)** | **P value** |
| --- | --- | --- | --- |
| **Pre-pregnancy BMI × GWG** | Normal/underweight BMI + Adequate GWG | Ref | – |
|  | Normal/underweight BMI + Inadequate GWG | 0.29 (0.00–2.54) | 0.321 |
|  | Normal/underweight BMI + Excessive GWG | 2.52 (0.96–6.99) | 0.061 |
|  | Overweight/obese BMI + Adequate GWG | 1.05 (0.34–3.17) | 0.935 |
|  | Overweight/obese BMI + Inadequate GWG | 1.39 (0.32–5.05) | 0.638 |
|  | Overweight/obese BMI + Excessive GWG | 3.80 (1.61–9.86) | 0.002 |
| **Infant sex** | Male vs Female | 1.82 (0.96–3.57) | 0.064 |
| **Gestational age at delivery (weeks)** | Continuous | 1.11 (0.79–1.58) | 0.532 |
| **Maternal age (years)** | Continuous | 1.02 (0.96–1.08) | 0.617 |

Notes:

Firth penalized logistic regression was performed to address quasi-complete separation in the combined ppBMI and GWG categories.

All models were adjusted for infant sex, gestational age at delivery, and maternal age.

Results were consistent with the primary analysis.

aOR = adjusted odds ratio; CI = confidence interval; Ref = reference category.
